# Dispersive Liquid-Liquid Microextraction of Bismuth in Various Samples and Determination by Flame Atomic Absorption Spectrometry

**DOI:** 10.1155/2016/6802646

**Published:** 2016-01-11

**Authors:** Teslima Daşbaşı, Şenol Kartal, Şerife Saçmacı, Ahmet Ülgen

**Affiliations:** ^1^Gemerek Vocational High School, Department of Food Technology, Cumhuriyet University, 58840 Sivas, Turkey; ^2^Department of Chemistry, Faculty of Sciences, Erciyes University, 38039 Kayseri, Turkey

## Abstract

A dispersive liquid-liquid microextraction method for the determination of bismuth in various samples by flame atomic absorption spectrometry is described. In this method, crystal violet was used as counter positive ion for BiCl_4_^−^ complex ion, chloroform as extraction solvent, and ethanol as disperser solvent. The analytical parameters that may affect the extraction efficiency like acidity of sample, type and amount of extraction and disperser solvents, amount of ligand, and extraction time were studied in detail. The effect of interfering ions on the analyte recovery was also investigated. The calibration graph was linear in the range of 0.040–1.00 mg L^−1^ with detection limit of 4.0 *μ*g L^−1^ (*n* = 13). The precision as relative standard deviation was 3% (*n* = 11, 0.20 mg L^−1^) and the enrichment factor was 74. The developed method was applied successfully for the determination of bismuth in various water, pharmaceutical, and cosmetic samples and the certified reference material (TMDA-64 lake water).

## 1. Introduction

Metal containing compounds have been used in medicine and health applications for a long time [1–3]. Bismuth is in the same group of the periodic table as nitrogen, phosphorus, arsenic, and antimony. The elements in this group are directly or indirectly concerned with the maintenance of human health as either essential elements or therapeutic/toxic elements. Conversely to other heavy metals, it is almost nontoxic, even though bismuth toxicity has been reported due to careless use [1–6]. Bismuth together with antibiotics (triple therapy) was suggested as a standard treatment for peptic ulcer and* Helicobacter pylori* infection, and it also has potential for the treatment of other diseases [5]. Furthermore, bismuth complexes have also been shown to exhibit promising antitumor activities [1–3].

For determination of bismuth in various samples, some instrumental techniques such as electrothermal atomic absorption spectrometry (ET-AAS) [7–11], inductively coupled plasma optical emission spectrometry (ICP-OES) [12–14], inductively coupled plasma mass spectrometry (ICP-MS) [15–18], hydride generation atomic absorption spectrometry (HG-AAS) [19–21], hydride generation atomic fluorescence spectrometry (HG-AFS) [22], electrothermal vaporization atomic fluorescence spectrometry (ETV-AFS) [23], hydride generation inductively coupled plasma atomic emission spectrometry (HG-ICP-AES) [24], potentiometric stripping analysis (PSA) [25], stripping voltammetry [26, 27], and flame atomic absorption spectrometry (FAAS) [28, 29] have been used.

However, ET-AAS and ICP-OES instruments are expensive and consume a lot of energy. In addition, these instruments are often not available in many laboratories. Hydride generation (HG) AAS and AFS are widely used techniques for the determination of elements As, Bi, Sb, and Se [30]. However, sampling procedure of this technique is more complicated and the costs for analysis are higher than direct FAAS determination. For many metal ions, direct FAAS determination is more useful and economical in trace analysis. However, direct determination of trace amounts of bismuth and some ions in several materials could be complicated, because the trace elements are present in various matrices (metals, mines, minerals, compounds, water, and organic and biological substances) and in levels of mg L^−1^ or *μ*g L^−1^. Due to insufficient sensitivity and matrix interference, the direct determination of the metal ions at trace levels, by this technique, is restricted. Thus, a separation and preconcentration step prior to the analysis is essential [14, 31]. The amount of trace elements or ultratrace elements could be measured sensitively, accurately, and successfully in these matrices by separation and preconcentration techniques with FAAS [28, 29].

Some preconcentration methods are used to analyze bismuth in various samples, such as solvent extraction [32–34], cloud point extraction (CPE) [9, 12, 30, 35], dispersive liquid-liquid microextraction (DLLME) [18, 36, 37], liquid-liquid extraction (LLE) [38], supported liquid membrane (SLM) [39], coprecipitation [40], and solid phase extraction (SPE) [11, 14, 20, 27, 28, 41].

In the present paper, a new dispersive liquid-liquid microextraction procedure has been reported for the determination of bismuth in various water, pharmaceutical, and cosmetic samples. In this procedure, crystal violet reagent (complexing agent) dissolved in ethanol (disperser solvent) containing chloroform (extraction solvent) is directly injected into the aqueous solution involving Bi(III) ions. The analyte in the sample solution is extracted into the fine droplets of extraction solvent. After the completion of extraction, the phase separation is performed by rapid centrifugation and Bi(III) ions enriched in the chloroform phase were determined by flame atomic absorption spectrometry (FAAS). The advantages of the DLLME method are simplicity of operation, rapidity, low cost, and high recovery and enrichment factors.

## 2. Experimental

### 2.1. Instrument

All absorbance measurements were carried out using a PerkinElmer (Norwalk, CT, USA) model AAnalyst 800 flame atomic absorption spectrometer equipped with a deuterium background corrector and an air-acetylene burner. A bismuth hollow cathode lamp from PerkinElmer served as the radiation source and was operated at a current of 20 mA. The wavelength of 223.1 nm with a 0.2 nm spectral bandpass and an acetylene/air flow rate of 2.9/17 L min^−1^ were used to as conventional working parameters. During the signal measurements, the integration time was 0.1 s. All pH measurements were made with a Consort C533 model digital pH meter equipped with a combined pH electrode, and a Hettich model Rotofix 32 centrifuge was used for the centrifugation of the ternary solvent system for the phase separation purpose.

### 2.2. Reagents and Solutions

All the reagents used were of the highest available purity or at least analytical reagent grade (Merck, Darmstadt, Germany). The laboratory glassware was kept overnight in a 1.4 mol L^−1^ HNO_3_ solution. Before use, all of the glassware was washed with deionized water and dried. Deionized water was used for the preparation of the solutions. A stock solution of bismuth at a concentration of 1000 *μ*g mL^−1^ was prepared by dissolving appropriate amount of Bi(NO_3_)_3_·5H_2_O in a 100 mL volumetric flask using 3 mol L^−1^ HNO_3_ to prevent hydrolysis of the Bi(III) ions. Working solutions were prepared by appropriate dilutions of the stock solution of Bi(III) with 0.1 mol L^−1^ HNO_3_ just before use [3, 42].

### 2.3. Dispersive Liquid-Liquid Microextraction Procedure

Before applying the developed DLLME method to real samples, it was optimized by using aqueous model solutions. To prepare the model solution, 1 mL deionized water, 50 *μ*L of 1% (w/v) crystal violet solution, and 43 *μ*L of 1.0 *μ*g mL^−1^ Bi(III) were placed into a centrifuge tube with a conical bottom and then the acidity of model solutions was adjusted to 0.05 mol L^−1^ HCl. A mixture was prepared from 300 *μ*L of chloroform (extraction solvent) and 1200 *μ*L of ethanol (disperser solvent) and then this mixture was rapidly injected into the sample solution. The final volumes were completed to 5 mL. The cloudy solution formed was centrifuged at 3500 rpm for 8 min, and the analyte in the sample was extracted into the extraction solvent. After the centrifugation, the organic phase was sedimented at the bottom of the centrifuge tube. The volume of final measurement solution was acquired approximately at 215 *μ*L. Then, a 50 *μ*L aliquot of this solution was rapidly introduced to the nebulizer of the flame atomic absorption spectrometer by using a microsyringe [43–45]. The bismuth signals were measured in the peak area mode utilizing the instrument software. The calibration graph was prepared against aqueous standards by submitting to the same DLLME method. A blank submitted to the same procedure was measured synchronically to the samples and calibration standards.

### 2.4. Analysis of Certified Reference Material (CRM)

The certified reference material (TMDA-64 lake water) was used to verify the accuracy of the developed method. 100 mL portions of TMDA-64 lake water were transferred into polyethylene bottles. To these sample solutions, 10 mL of concentrated HNO_3_ (65%, w/w) and 5 mL of concentrated H_2_O_2_ (30%, w/w) were added and heated to near dryness on a hot plate. Finally, the residues were taken with deionized water and the acidity of sample solutions was adjusted to 0.05 mol L^−1^ HCl. The final volumes were completed to 5 mL. Then the DLLME procedure was applied to these treated CRM samples. The determination of Bi(III) ions in the final solutions was performed by FAAS.

### 2.5. Application of the Proposed Method to Real Samples

The proposed method was successfully applied for the determination of Bi(III) in tap water, dam water (Yozgat), waste water (Kayseri), pharmaceutical, and cosmetic samples. The water samples were collected in prewashed polyethylene bottles. 100 mL of the water samples was transferred into 250 mL beakers. 10 mL of concentrated HNO_3_ (65%, w/w) and 5 mL of concentrated H_2_O_2_ (30%, w/w) were added to 100 mL of water samples and heated to near dryness on a hot plate. The resulting clear solution was diluted to 5 mL with deionized water. The acidity of sample solutions was adjusted to 0.05 mol L^−1^ HCl. The proposed DLLME procedure was applied to these sample solutions and Bi(III) ions in the enriched phase were determined by FAAS. The blank solutions were prepared in the same manner without the addition of bismuth ions and the final solutions were measured by FAAS.

Additionally, creams as pharmaceutical sample and hair dyes (black, brown color) as cosmetic products were analyzed for determination of Bi(III) by FAAS using the proposed DLLME procedure. 2 g of the hemorrhoid cream (1), 4 g of the hemorrhoid cream (2), and 5 g of the burn cream samples were separately taken into 100 mL beakers. 10, 20, and 25 mL aliquots of concentrated HNO_3_ (65%, w/w) were added to the cream (1), cream (2), and burn cream samples, respectively. The samples were heated to near dryness on a hot plate in hood. Then, 4, 8, and 10 mL aliquots of concentrated HClO_4_ (70%, w/w) were added to the cream (1), cream (2), and burn cream residues, respectively. After the evaporation, the cooled residues were dissolved and made up to 5 mL with deionized water. For the analysis of hair dye samples, 5 g aliquots were transferred into the beakers. The dissolving process was continued as just mentioned above (using 20 mL of concentrated of HNO_3_ (65%, w/w) and 8 mL of concentrated HClO_4_ (70%, w/w)). The resulting clear solution was diluted to 5 mL with deionized water. The acidity of the sample solutions was adjusted to 0.05 mol L^−1^ HCl. The developed DLLME procedure was applied to these sample solutions and blanks prepared in the same way. Finally, the solutions were measured by FAAS.

## 3. Results and Discussion

### 3.1. Effect of Acidity of Solution

**Figure 1:**
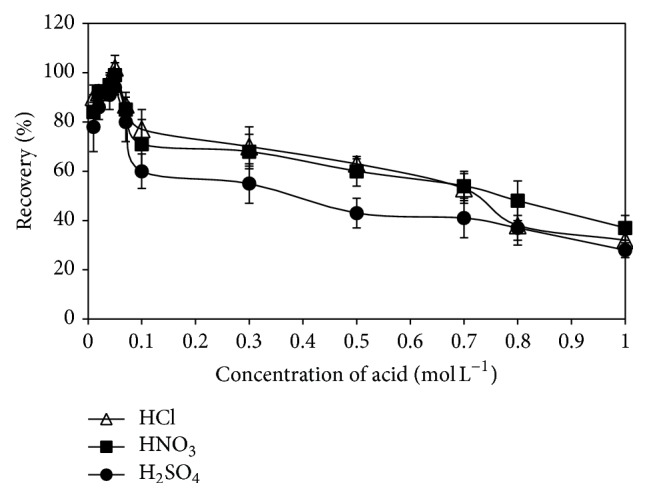
Effect of the acidity of the sample solution on the recovery of Bi(III) ions by using the proposed DLLME procedure.

Acidity of the sample solution plays a unique role in metal-chelate formation and its subsequent extraction in most of analytical processes. Sample solutions were acidified from 0.01 to 1.0 mol L^−1^ with HCl, HNO_3_, and H_2_SO_4_ and processed according to the recommended procedure. The effects of concentration and type of acid on the recovery efficiency of the DLLME procedure for the determination of bismuth are shown in Figure 1. The percent recoveries of bismuth as a function of the acidity were increased from 0.01 to 0.05 mol L^−1^ for the three acids. It was observed that the extraction yield for bismuth was decreased over 0.05 mol L^−1^ acid concentration in a stepwise manner. When the acidity of a solution is lower than 0.01 mol L^−1^ HNO_3_, the absorbance of bismuth decreased because of the hydrolysis of Bi(III) ions in this low acidic medium [7]. Bi(III) can only exist in very acidic media and it is hydrolyzed to BiOH^2+^ or BiO^+^ in acidic media ≤0.1 mol L^−1^. At these low acidic media, even insoluble compounds such as BiOCl or BiONO_3_ could be formed [9]. For this reason, the optimum acidity of the sample solutions was chosen to be 0.05 mol L^−1^ HCl throughout the experiments.

### 3.2. Effect of the Amount of Crystal Violet

**Figure 2:**
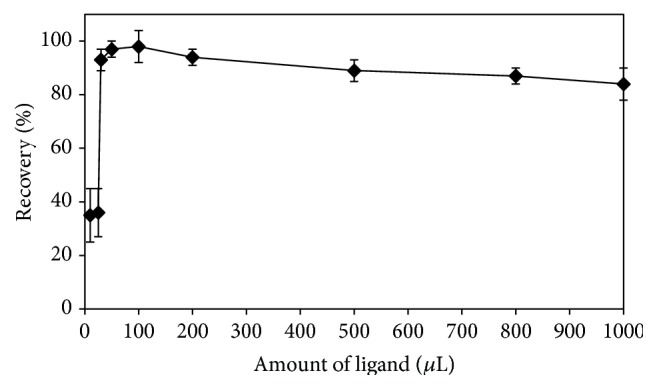
Effect of the amount of 1% crystal violet on the recovery of Bi(III) ions.

Choosing the complexing agent to be complexed with the metal ions plays a fundamental role in the separation of traces of the analyte from the aqueous phase to the organic phase in the DLLME processes. By considering this significance of the reagent, crystal violet (CV) was selected to form BiCl_4_^−^CV^+^ ion-association pair [46]. The influence of the ligand amount on the recovery efficiency of bismuth(III) ions was studied in the range of 10–1000 *μ*L of 1% (w/v) crystal violet solution (see Figure 2). The recoveries were quantitative at quantities more than 30 *μ*L of 1% (w/v) solutions of the ligand. Therefore, a 50 *μ*L aliquot of 1% (w/v) crystal violet solution was selected for further studies.

### 3.3. Selection of Disperser Solvent

Choosing the most suitable disperser solvent is of primary importance to achieve good selectivity of the analyte. The disperser solvent must be miscible with both extraction solvent (organic phase) and sample solution (aqueous phase). For the selection of the best appropriate disperser solvent, methanol, ethanol, acetone, and acetonitrile were tested in the DLLME process. The values of recoveries were calculated to be 92 ± 8% with methanol, 102 ± 4% with ethanol, 66 ± 5% with acetone, and 84 ± 6% with acetonitrile. Therefore, chloroform as an extraction solvent was selected for further experiments. In these experiments, 300 *μ*L of CHCl_3_ as the extraction solvent and 1200 *μ*L of each disperser solvent were used. The maximum recovery of bismuth(III) was obtained by using ethanol as a disperser solvent.

### 3.4. Selection of Extraction Solvent

The selection of an appropriate extraction solvent is of significance to succeed in good selectivity of analytes. In this study, extraction solvent used had a density higher than that of water [47, 48]. A series of sample solutions were studied by using 1200 *μ*L of ethanol and 300 *μ*L of different extraction solvents, which are chloroform, dichloromethane, and carbon tetrachloride. The highest recovery value of bismuth for the proposed DLLME method was obtained with chloroform. Quantitative recoveries and low standard deviation were achieved with this solvent. The values of recoveries were calculated to be 100 ± 3% with chloroform, 85 ± 5% with dichloromethane, and 71 ± 4% with dichloromethane. Therefore, chloroform as an extraction solvent was selected for further experiments.

### 3.5. Effect of Volume of Extraction Solvent

**Figure 3:**
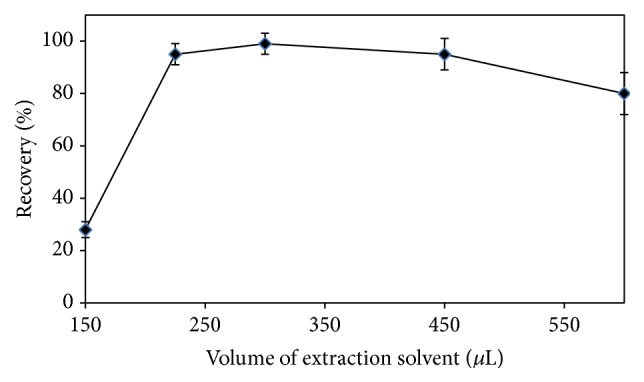
Effect of the volume of chloroform on the extraction efficiency of the DLLME method.

In order to investigate the effect of the ratio of the extraction solvent on the disperser solvent, a series of sample solutions were prepared by mixing the different amounts of chloroform, that is, 150, 225, 300, 450, and 600 *μ*L with 1200 *μ*L of ethanol in a centrifuge tube, and then the effect on the recovery efficiency of the DLLME method was tested. The results attained are shown in Figure 3. The quantitative recoveries were achieved in the range of 225 to 450 *μ*L of chloroform. As can be seen from the results, the highest recovery was obtained with a mixture containing 300 *μ*L of chloroform and 1200 *μ*L of ethanol.

### 3.6. Effect of the Total Volume of the Organic Phase

**Figure 4:**
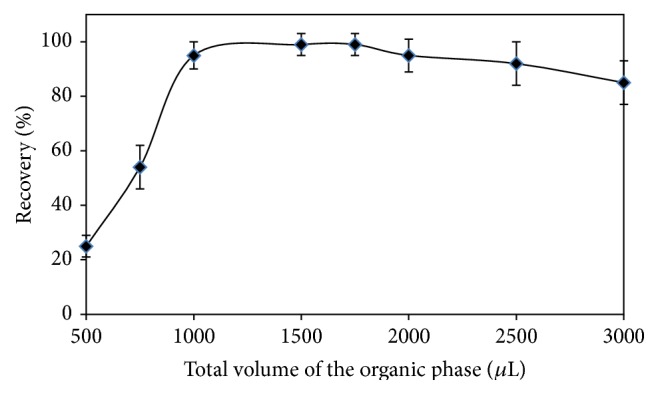
Effect of the total volume of the organic phase on the recovery of bismuth.

In the proposed DLLME process, ethanol as the disperser solvent and chloroform as the extraction solvent were used. The effect of total volume of the organic solvents on the extraction efficiency was studied by using increasing total volume of the organic solvents ranging between 0.5 and 3.0 mL under the optimum experimental conditions. The results are shown in Figure 4 and the recoveries (%) were quantitative in the range of 1.0–2.0 mL. As the optimum total volume of the organic solvents, 1.5 mL was selected throughout the experiments, that is, 300 *μ*L CHCl_3_ and 1200 *μ*L C_2_H_5_OH.

### 3.7. Effect of Centrifugation Variables

**Figure 5:**
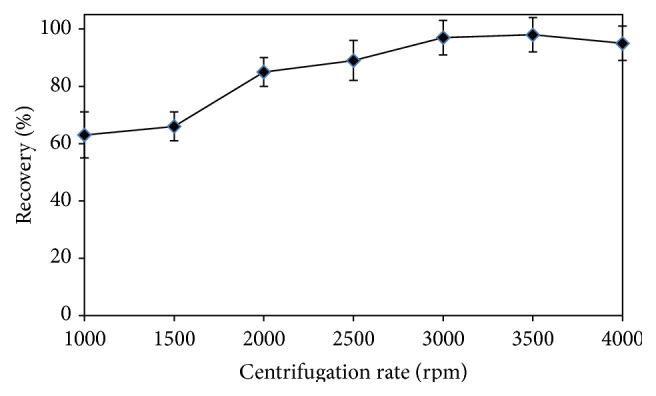
Effect of centrifugation rate (rpm) on the recovery (%) of the DLLME procedure for Bi(III) ions.

**Figure 6:**
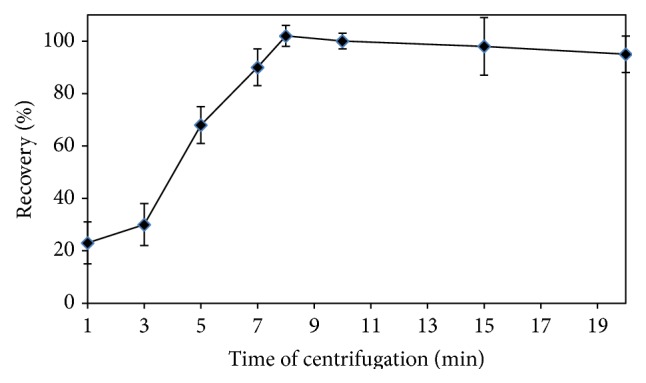
Effect of the centrifugation time on the extraction efficiency of the DLLME extraction procedure for Bi(III) ions.

According to other extraction methods, comparatively, one of the main advantages of DLLME procedure is that a shorter extraction time is required, as a result of the infinitely large surface area formed between extractant and aqueous phase. Determining of the extraction time plays an important role in the efficiency of the method. The centrifugation rate was scanned from 1000 to 4000 rpm. During the scanning, the centrifugation time was kept constant to be 8 min, and the results are shown in Figure 5. As can easily be seen from Figure 5, the optimal centrifugation rate was selected as 3500 rpm. Afterwards, the effect of centrifugation time on the extraction efficiency was examined. Therefore, in order to get the best extraction time in the centrifugation process, it was studied in the time period of 1 to 20 min at the fixed centrifugation rate of 3500 rpm. According to the results given in Figure 6, the quantitative recoveries were achieved in the period of 8 to 20 min as centrifuging time. The most time-consuming step was the centrifuging time in the proposed DLLME method, for which the optimal value was 8 min.

### 3.8. Effect of Foreign Ions

The efficiency of the developed DLLME method for the preconcentration of bismuth in the presence of various cations and anions was examined. To perform this study, different amounts of foreign ions were added to model solutions containing 0.20 mg L^−1^ Bi(III) and were subjected to the recommended procedure. The tolerance limit of the foreign ions is defined as the largest amount of interfering ions causing less than ±5% relative error in the recovery values of Bi(III) ions. The results related to the interference effects of important alkali, earth alkaline, and some transition metal ions including aluminum, lead, sulfate, and dihydrogenphosphate are summarized in Table 1. As can be seen from Table 1, there was not any observed interference effect from the studied anions and cations at the interferant concentrations changing from 50 (for Al^3+^) to 3000 mg L^−1^ (for Na^+^).

### 3.9. Accuracy and Applications of the DLLME Method

In order to establish the accuracy of the proposed procedure, the method has been applied to the certified reference material (TMDA-64 lake water) for the determination of bismuth. The results obtained by the proposed preconcentration method (0.140 ± 0.007 mg L^−1^) and certified value (0.142 ± 0.028 mg L^−1^) are in very good agreement (*n* = 5).

After verifying the accuracy of the proposed method, it was applied to real samples for the determination of Bi(III) ions in various water, pharmaceutical, and cosmetic samples. The obtained results are shown in Table 2. The recovery studies were carried out with the real samples spiked with bismuth in accordance with their bismuth levels. According to this table, the added bismuth ion can be quantitatively recovered from the samples by the developed DLLME procedure. The recoveries for the additions of different amounts of Bi(III) varied from 95 to 101%. These results prove the accuracy of the proposed method. These recovery values indicate that the efficiency of the proposed DLLME method is very good with adequate standard deviation.

### 3.10. Analytical Figures of Merit

For the purpose of quantitation of the analyte, a calibration curve was obtained under the optimum conditions by applying the proposed method. Primarily, the standard solutions were prepared in the range of 0.040–1.00 *μ*g mL^−1^ Bi(III) and then the absorbances were measured by FAAS in the peak area mode utilizing the software of the instrument. The calibration curves for both aqueous standards and standards in chloroform, which were obtained by using the least squares method, have determination coefficients (*R* ^2^) of 0.9991 and 0.9988 and the calibration equations were as follows: *A* = −0.0005 + 0.0646 × *C*_Bi_ and *A* = −0.0008 + 0.0661 × *C*_Bi_, respectively, where *A* is absorbance and *C*_Bi_ is the concentration (*μ*g mL^−1^) of Bi(III) ions. The slopes and the absorbances obtained for each individual standard solution of the two calibration curves are in a good agreement with each other. The results obtained showed that the only difference was the color change in the two flames of the aqueous calibration standards and the standards present in chloroform phase. The detection limit of the method (DL) was 4.0 *μ*g L^−1^ Bi(III) (*n* = 13). In order to measure the precision of the method, the model solutions containing 0.20 *μ*g mL^−1^ Bi(III) (*n* = 11) were prepared, the proposed method was applied to these sample solutions, and the relative standard deviation (RSD) was calculated to be 3%. Enrichment factor (EF) is a measure of sensitivity of the method. Sample volume has a unique role to achieve high EF and depends on the amount of extracting phase. In order to deal with real samples, especially water samples, containing very low concentrations of the bismuth ions, the maximum applicable sample volume was determined. For this purpose, the developed method was applied to 5–20 mL volumes of the model solutions containing 0.20 *μ*g mL^−1^ Bi(III), under the optimum conditions. The preconcentration factor was calculated as the ratio of the highest sample volume (≈16 mL) to the volume of final measurement solution (≈215 *μ*L) and found to be 74 for bismuth(III) ions.

A comparison of the described method with the other preconcentration methods for bismuth is given in Table 3. The results of this study indicate that the developed DLLME procedure combined with flame atomic absorption spectrometry for the determination of trace levels of bismuth in various samples was fully successful.

The DL of proposed method was the same as reported by Wen et al. [29]. However, preconcentration factor of this study was higher than those reported by Wen et al. [29], by Afkhami et al. [35], and by Fayazi et al. [36] (Table 3). In these two studies, the concentration of bismuth was determined by FAAS.

## 4. Conclusions

In the present study, the proposed DLLME procedure combined with FAAS has been used for the determination of bismuth in various water, pharmaceutical, and cosmetic samples. This method offers several advantages including being inexpensive, rapid, and safe and having lower toxicity, low sample consumption, low contamination, good recovery, and high enrichment factor. The proposed method was applied to the certified reference material (TMDA-64 lake water) and spiked and unspiked real samples. The recoveries for the additions of different amounts of Bi(III) varied from 95% to 101%. High preconcentration factor was obtained easily through this method and a detection limit at *μ*g L^−1^ level was achieved with only 5.00 mL of sample. Although the obtained results in this work are related to bismuth determination, the system could be readily applied to the determination of other metals using various ligands, extractable by other organic solvents. This procedure has been demonstrated to be promising for trace bismuth analysis, due to its high tolerance to interference from the matrix.

## Figures and Tables

**Table 1:** Effect of foreign ions on the determination of 0.2 mg L^−1^Bi(III) using the DLLME proposed procedure (*n* = 3).

Ions	Added as	Concentration (mg L^−1^)	Recovery (%)
Na^+^	NaNO_3_	3000	98 ± 5^a^
K^+^	KNO_3_	1000	103 ± 3
Fe^3+^	Fe(NO_3_)_3_·6H_2_O	1000	102 ± 4
Ca^2+^	Ca(NO_3_)_2_·4H_2_O	1000	97 ± 4
Mg^2+^	Mg(NO_3_)_2_·6H_2_O	1000	95 ± 3
Zn^2+^	Zn(NO_3_)_2_	100	98 ± 4
Cr^3+^	Cr(NO_3_)_3_·9H_2_O	100	100 ± 3
Cu^2+^	Cu(NO_3_)_2_·4H_2_O	100	97 ± 5
Mn^2+^	Mn(NO_3_)_2_·4H_2_O	100	97 ± 3
Cd^2+^	Cd(NO_3_)_2_·4H_2_O	100	99 ± 2
Co^2+^	Co(NO_3_)_2_·4H_2_O	100	97 ± 3
Pb^2+^	Pb(NO_3_)_2_	70	96 ± 4
Al^3+^	Al(NO_3_)_3_·9H_2_O	50	96 ± 4
SO_4_^2−^	Na_2_SO_4_	1000	102 ± 4
H_2_PO_4_^−^	NaH_2_PO_4_·2H_2_O	1000	100 ± 1

^a^Average ± standard deviation.

**Table 2:** The determination of bismuth in water, cream, and hair dye samples (*n* = 3).

Sample	Added	Found	Recovery (%)
Dam water, Yozgat (*µ*g L^−1^)	—	—	—
	100	98 ± 3^a^	98 ± 3
Waste water, Kayseri (*µ*g L^−1^)	—	—^b^	—
	100	96 ± 2	96 ± 2
Hair dye, black (*µ*g kg^−1^)	—	64 ± 3	—
	50	115 ± 13	101 ± 4
Hair dye, brown (*µ*g kg^−1^)	—	83 ± 4	—
	50	127 ± 14	95 ± 5
Hemorrhoid cream (1) (for analgesic) (*µ*g g^−1^)	—	3.3 ± 0.1	—
	1.5	4.8 ± 0.2	100 ± 2
Hemorrhoid cream (2) (*µ*g kg^−1^)	—	104 ± 18	—
	100	202 ± 17	99 ± 3
Burn cream (*µ*g kg^−1^)	—	98 ± 12	—
	100	191 ± 13	96 ± 4

^a^Average ± standard deviation.

^b^Below the detection limit.

**Table 3:** Comparison of the characteristic data between recently published works for bismuth.

Enrichment method	Using reagent	System	DL (*µ*g L^−1^)	RSD (%)	PF	Ref.
CPE	Dithizone, Triton X-114	ET-AAS	0.02	4.3, *n* = 5, 0.3 *µ*g L^−1^	196	[9]
CPE	8-Hydroxyquinoline, Triton X-114	FI-ICP-AES	0.12	2.3, *n* = 7	81	[12]
CPE	Bromopyrogallol red (BPR) Triton X-114	UV-VİS	2	2.41, *n* = 6, 50 *µ*g L^−1^	20	[35]
RS-CPE	Dithizone, Triton X-100, and octanol	FAAS	4	4.2, *n* = 11, 500 *µ*g L^−1^	43	[29]
Sequential injection	Methylthymol blue (MTB)	Spectrophotometer	250	1.1, *n* = 12, 50 mg L^−1^	—	[40]
FI-LLE	Tetraphenylarsonium chloride, chloroform	UV-VİS	1.5·10^−6^ M	1.6, *n* = 12, 3·10^−5^ M	—	[37]
DLLME	2-(5-Bromo-2-pyridylazo)-5-(diethyl amino) phenol, acetone, and dichlorobenzene	FAAS	3	1.5, *n* = 7, 0.4 mg L^−1^	28	[36]
DLLME	Crystal violet, ethanol, and chloroform	FAAS	4	3, *n* = 11, 0.2 mg L^−1^	74	This work

PF: preconcentration factor, DL: detection limit, RSD: relative standard deviation, Ref.: reference, ET: electrothermal, ICP-AES: inductively coupled plasma atomic emission spectrometry, FI: flow injection, RS: rapidly synergistic.
